# How Can Job Crafting Be Reproduced? Examining the Trickle-Down Effect of Job Crafting from Leaders to Employees

**DOI:** 10.3390/ijerph17030894

**Published:** 2020-01-31

**Authors:** Xun Xin, Wenjing Cai, Wenxia Zhou, Sabrine El Baroudi, Svetlana N. Khapova

**Affiliations:** 1 Business School, Southwest University of Political Science & Law, Chongqing 401120, China; xinxun0629@163.com; 2 School of Public Affairs, University of Science and Technology of China, Hefei 230026, China; 3 Department of Management and Organization, Vrije Universiteit Amsterdam, 1081HV Amsterdam, The Netherlands; s.elbaroudi@vu.nl (S.E.B.); s.n.khapova@vu.nl (S.N.K.); 4 School of Labor and Human Resources, Renmin University of China, Beijing 100872, China

**Keywords:** leader-subordinate, job crafting, job resources, empowering leadership, trickle-down effect

## Abstract

This study seeks to examine how and when job crafting trickles down from leaders to followers in a team context. Drawing on social learning theory, we hypothesize that team leaders’ job resources mediate the relationship between team leaders’ job crafting and team members’ job crafting. Empowering leadership is proposed to strengthen the mediation effect, such that under a stronger (higher) empowering leadership style the relationship between team leaders’ job resources and team members’ job crafting is further strengthened, thereby positively influencing the overall mediated relationship. We tested our multilevel moderated mediation model with leader-subordinate paired data from 64 work teams in seven Chinese enterprises over two time periods. The results support our hypothesized mediated relationship; however, contrary to our prediction, we find that empowering leadership negatively moderates the relationship between team leaders’ job resources and team members’ job crafting, and weakens the mediation effect of team leaders’ job resources. Theoretical and practical implications are discussed.

## 1. Introduction

Job crafting has garnered considerable research attention over the past decade [1,2,3] and has been found to have a significant impact on several employee outcomes, as well as on team and organizational outcomes [4,5]. Job crafting refers to a proactive behavior that is initiated by employees to alter the task and relational boundaries of work for career and work-related purposes [6,7]. Indeed, a plethora of empirical evidence suggests that job crafting is positively related to employee performance and wellbeing [8,9], team performance [9], and organizational change [10]. It is therefore not surprising that both academics and practitioners seek to better understand how job crafting can be enhanced in organizations.

In an attempt to further examine how employee job crafting can be facilitated, studies have started to recognize leadership as an important contextual predictor of the behavior. For instance, Wang, Demerouti, and Le Blanc [11] demonstrated that transformational leadership stimulates employee job crafting by increasing employee adaptability. Likewise, servant leadership was found to encourage employee job crafting, which in turn increased employee engagement [7]. Extending the focus to teams, Harju, Schaufeli, and Hakanen [6] found that servant leadership reduces job boredom by boosting job crafting behaviors in teams. While these findings make important contributions to the literature on leadership and job crafting, they neglect the fact that leaders may also encourage employee job crafting by role modeling the behavior. This transmission effect from leaders to lower-level employees is called the trickle-down effect and has been extensively studied in relation to other topics such as work engagement [12], ethical work behavior [13], and deviant work behavior [14], but has so far been largely neglected in job crafting research.

**Figure 1:**
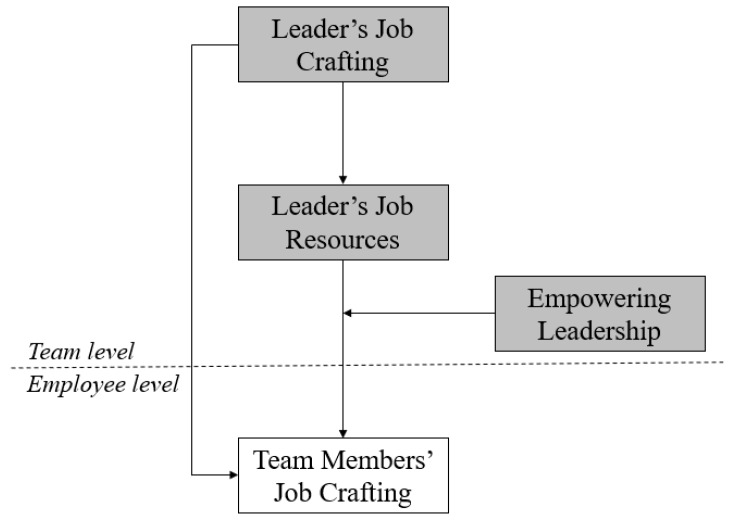
The hypothesized model.

The purpose of this study is to address this research gap in the context of teams. Prior recent work has shown that the trickle-down effect is strongly prevalent in teams [6,7,12], and because job crafting occurs more often in the context of teams [5,6,7], it is necessary to understand whether and how leader job crafting behavior can be transmitted to team members. In line with research on the trickle-down effect, we use social learning theory as the theoretical foundation of our arguments [12,13]. According to social learning theory [15,16], people learn values, emotions, attitudes, and behaviors from leaders. It is therefore likely that leader job crafting may be imitated and learned by followers. In line with prior work examining the trickle-down effect in teams [12], we suggest that job crafting behavior will be most likely transmitted from team leaders to team members, because team leaders are closer to members and team members can therefore more easily imitate their behavior. We further suggest that the job resources of a team leader will mediate the positive relationship between team leader’s job crafting and team members’ job crafting, especially because job crafting research demonstrates that individuals engage in job crafting to gain structural (i.e., autonomy and opportunities for development) and social (i.e., social support) job resources [5,6,7,9]. Team members will thus understand that job crafting produces positive outcomes; according to social learning theory, this is why they will mimic their leader’s behavior [15,16]. Finally, we propose that empowering leadership will moderate the mediated relationship, because a leader who encourages followers to engage in job crafting tends to adopt an empowering leadership style [17], thereby further enhancing job crafting behaviors in teams. Figure 1 displays our multilevel moderated mediation model.

The current study aims to make a threefold contribution to the job crafting literature. First, we extend research on job crafting that takes a leadership approach by introducing a trickle-down effect of job crafting from leaders to followers. This is the first study to examine such a transmission effect in a team context and provides an explanation for why this effect occurs (i.e., mediation effect of leader’s job resources), thereby contributing to a more comprehensive understanding of why leadership facilitates employee job crafting behavior. Second, by examining empowering leadership as a moderating variable that influences the trickle-down effect, we further clarify the conditions under which job crafting can be transmitted from leaders to followers; thus, this study is the first to demonstrate that multiple leadership elements may jointly impact the transmission effect. Third, by introducing social learning theory to job crafting research, we attempt to enrich theoretical perspectives on job crafting in the current literature that largely focus on the job-demands-resources theory [4,18]. By doing so, we respond to research calls to more explicitly reveal the social learning process that underlies the trickle-down effect [19].

## 2. Literature Review and Hypotheses

### 2.1. Job Crafting

Job crafting refers to an individual’s proactive efforts to make physical and cognitive changes in their jobs to create a better fit between job demands and personal needs [20]. Due to better job fit, several positive work outcomes for employees can be achieved including job satisfaction, performance, and work engagement [3,21,22,23,24,25]. Cognitive changes are not considered to be behavioral changes, but alternations in how employees view their work. For instance, work may be viewed as a set of discrete parts or as an integrated whole, and employees may change their perceptions about the importance of their work to others [26]. On the other hand, since the cognitive changes pay more attention to individuals’ inner heart which cannot be easily changed and will not cause any actual changes to the job [26], scholars are increasingly highlight that physical changes are behavioral and are made in job tasks or in the social work environment [4,27]. We, in the current study, follow this line of research to define job crafting as behaviors only involving in changing task and relational boundaries. Task alternations are performed, for instance, to increase or decrease the number of activities one is involved in at work, such as minimizing the negative aspects of work and/or increasing its positive aspects. Relational alternations involve exercising discretion over one’s social interactions at work by changing the quality and/or amount of interaction with others at work. For instance, employees may proactively decide to interact more with certain colleagues to receive feedback [24,25]. While the positive outcomes of job crafting for employees have largely been examined [3,21,22,23,24,25], it has recently been acknowledged that the behavior improves team functioning [28,29].

### 2.2. Trickle-Down Effect of Job Crafting: From Team Leaders to Team Members

Researchers have used the word “trickle-down” to describe the transmission of attitudes and behaviors from supervisors to subordinates [30], using the social learning theory as the predominant theoretical underpinning to explain the transmission effect [12,13,14]. According to the theory, those who are lower in the hierarchy tend to emulate and imitate the behavior of their supervisor, because the latter is commonly considered to be a legitimate role model, especially because of his/her higher status over those at lower organizational levels [15,16,18,30]. Furthermore, the theory posits that when a leader is proximate to followers, his/her behavior becomes highly visible to them making it easier to imitate the leader’s behavior [12,15,16,31]. Leaders tend to engage in job crafting behaviors [32] and because the connection between leaders and team members is stronger in a team context, it is likely that team members will imitate a team leader’s job crafting behavior. Studies have demonstrated that in a team context, leaders engaged in other (related) self-initiated work behaviors such as helping behaviors, which were transmitted to followers in teams [33,34]. Based on the social learning theory and empirical evidence, we predict that a team leader’s job crafting behavior will be imitated by team members. We therefore hypothesize the following:

**Hypothesis 1** **(H1):** Team leaders’ job crafting is positively related to team members’ job crafting.

### 2.3. Mediating Effect of Leaders’ Job Resources

Social learning theory further posits that individuals choose to enact a behavior learned from role models based on the perceived positive consequences of that behavior [15]. Therefore, job crafting behavior will be imitated by team members when they perceive that it produces positive outcomes. According to job crafting literature individuals engage in job crafting behaviors to accumulate job resources to facilitate performance or reduce job demands and stress [35,36,37,38,39]. For instance, Tims, Bakker, and Derks [9] demonstrate that when employees alter their tasks, they do so for the purpose of gaining autonomy, task variety, challenges, and learning opportunities at work, which increases their structural job resources (i.e., autonomy and opportunities for development). When employees alter their relations at work, they do so to seek feedback from colleagues and supervisors, or to increase their professional network which leads to more social job resources (e.g., social support at work) [9]. A team leader who engages in job crafting behavior will thus increase his/her job resources. Because teamwork is characterized by shared responsibilities and common goals [40], team leaders will likely feel responsible for improving team performance and will thereby share their job resources with their team [41,42]. Thus, in a team context, team members will understand that there are more job resources available because their team leader engages in job crafting, which in turn will likely encourage them to imitate the leader’s job crafting behavior. Accordingly, we predict that team leaders’ job crafting enhances their job resources, which, in turn, positively influences team members’ job crafting. Therefore, we hypothesize the following:

**Hypothesis** **2 (H2):** Team leaders’ job resources mediate the trickle-down effect of job crafting from team leaders to team members.

### 2.4. The Moderating Role of Empowering Leadership

Since leadership style is aligned with a leader’s attitudes and behaviors, a leader who engages in job crafting and encourages his/her followers to engage in the behavior will adopt a leadership style that matches the proactive behavior, which is an empowering leadership style [17]. Empowering leadership involves behaviors such as sharing power and authority, encouraging followers to take more responsibility, cultivating followers’ self-leadership skills, enhancing followers’ confidence to improve performance, and supporting followers’ development through coaching, role modeling, and feedback [43,44,45]. However, since team leaders have other supervisors who are higher in the hierarchy, they will only be able to successfully adopt an empowering leadership style if they are supported in doing so. We therefore treat empowering leadership as a moderating contextual factor, which may be highly or weakly adopted in a team context. Furthermore, we propose that empowering leadership will moderate the relationship between team leaders’ job resources and team members’ job crafting, especially because team leaders may more easily adopt an empowering leadership style when they have accumulated structural and social job resources (i.e., autonomy and social support). When team leaders can adopt a stronger empowering leadership style, they will offer team members more opportunities such as autonomy and confidence to engage in job crafting behaviors [43,44,45], thereby strengthening the positive relationship between team leaders’ job resources and team members’ job crafting. We expect this moderating effect of empowering leadership style to be weaker when the leadership style is weakly adopted. Therefore, we hypothesize the following:

**Hypothesis** **3 (H3):** Empowering leadership moderates the relationship between team leaders’ job resources and team members’ job crafting, such that this relationship is stronger among leaders with high empowering leadership than among those with low empowering leadership.

Furthermore, social learning theory argues that followers will more likely mimic their leader’s behavior if they are rewarded for imitating the behavior [15,16]. Empowering leadership style may be viewed as a reward because it signals to team members that their team leader appreciates their job crafting behavior. Under this leadership style, team members may be more strongly encouraged to imitate their leader’s job crafting behavior. We thus hypothesize a moderated mediation effect between team leaders’ job crafting, team leaders’ job resources, team members’ job crafting, and empowering leadership. Specifically, we predict that leaders’ job crafting is positively associated with team members’ job crafting through the mediating effect of team leaders’ job resources. This mediated relationship is proposed to be further strengthened, because of the positive moderation effect of empowering leadership in the path between team leaders’ job resources and team members’ job crafting. The mediation effect is stronger when empowering leadership is high than when it is low. Therefore, we hypothesize the following:

**Hypothesis 4** **(H4):** Empowering leadership moderates the mediated relationship between team leaders’ job crafting and team members’ job crafting via leaders’ job resources, such that the mediated relationship is stronger among leaders with high empowering leadership than among those with low empowering leadership.

## 3. Materials and Methods

### 3.1. Sample and Procedures

The data collection was conducted in seven high-tech companies in Beijing, P.R. China in two stages. In the first stage, we used our personal network to contact the enterprises and asked for their interest in participating in the project. Seven enterprises accepted our invitation and we sent our survey to the heads of the human resource department to obtain their approval for its content. After receiving approval, the heads of the human resource departments decided which work teams could participate in our study and sent us a list with contact information for all participants. The work teams consisted of team leaders and team members who had worked together for at least one year. Each work team had one leader and three to eight team members. In the second stage, we started collecting the data. Surveys were sent by mail to team leaders and we asked the team leaders to distribute the surveys to their team members. This strategy is in line with previous research examining work behaviors in teams [46,47]. To enhance the rigor of our research design, we collected data at two different time points with a two-month interval between each point. At Time 1, the surveys were distributed to a sample of 560 individuals, including 100 team leaders and 460 team members. The leaders of each team rated their own job crafting and job resources, and the team members rated their leader’s leadership style (i.e., empowering leadership). At Time 2, another survey was distributed to the team members who were asked to rate their own job crafting behavior. The use of data with input from multiple sources decreases the possibility of common method bias. Through two rounds of data collection, 64 usable leader-subordinate matched samples were received, consisting of 64 team leaders and 250 team members (64 teams). The loss rate of the leader sample was 36%, and that of the team members sample was 46%.

The number of team members per team ranged from three to eight (M = 3.91). In the team members sample, most participants were male (64.2%), and the average age was 29.95 years (SD = 7.04). These participants had an average work experience of 5.84 years (SD = 7.26) in their employing organization. Of the respondents, 2% had an education below high school, 6% had a high school education, 6% had an associate’s degree, 62.6% had a bachelor’s degree, and 8.4% had at least a master’s degree. The participants were employed in a range of occupational sectors, including management departments (19%), administration (8.9%), technical departments (22.8%), research & development (R&D) departments (23.2%), business departments (8.9%), and production departments (17.3%).

In the team leader sample, most participants were also male (73.3%), and the average age was 37.35 years (SD = 6.99). These participants had an average work experience of 12.14 years (SD = 8.35) in their employing organization. Of the respondents, 3.2% had a high/secondary (or lower) education, 23.8% had a vocational education, 60.3% had a bachelor’s degree and 12.7% had at least a master’s degree. More than half of the participants were basic level managers (57.2%), some participants were middle-level managers (38.1%), and the remaining participants were top managers (4.8%). The team leaders led teams from different fields, including production (14.1%), marketing (18.8%), technology development (34.4%), production management (7.8%), and administrative personnel (26%).

### 3.2. Measures

We followed the commonly used back-translation procedure to translate all scales from English to Chinese [48].

Job crafting. Team leaders’ and team members’ job crafting was assessed using the expansion-oriented job crafting scale [49]. This scale consists of two subscales, i.e., physical and relational job crafting. The respondents were asked to provide responses on a 5-point Likert scale ranging from “not at all” to “very much so”. A sample item of task crafting is “I have taken steps to increase the challenges I am facing in my job”. A sample item of relational crafting is “I have taken steps to increase the extent to which I deal with other people on my job”. Cronbach’s alpha ranged from 0.88 to 0.92.

Leaders’ job resources. To examine the team leaders’ job resources, autonomy, social support, and opportunities for development were measured. The overall scale consisted of 13 items. Autonomy was assessed using a three-item scale [50]. A sample item is “Do you have flexibility in the execution of your job?” Social support was measured using two sub-scales: a three-item scale assessing social support [51] and a 3-item scale assessing leader direction [52]. Sample items of the two sub-scales are “If necessary, can you ask your colleagues for help?” and “My supervisor uses his or her influence to help me solve my problems at work.” Opportunities for development were measured using a four-item scale [50]. A sample item is “My work offers me the opportunity to learn new things.” The participants rated their responses on a 5-point Likert scale ranging from “Strongly disagree” to “Strongly agree”. Cronbach’s alpha ranged from 0.87 to 0.92.

Empowering leadership. To measure empowering leadership, we used the 12-item scale developed by Ahearne, Mathieu and Rapp [43]. The scale contains four dimensions that focus on enhancing the meaningfulness of work (three items), fostering participation in decision making (three items), expressing confidence in high performance (three items), and providing autonomy from bureaucratic constraints (three items). Sample items of each dimension are “My manager helps me understand how my objectives and goals relate to those of the company” (enhancing the meaningfulness of work), “My manager makes many decisions with me” (fostering participation in decision making), “My manager believes that I can handle demanding tasks” (expressing confidence in high performance), and “My manager allows me to do my job my way” (providing autonomy from bureaucratic constraints). Items were scored on a 6-point Likert scale ranging from “Strongly disagree” to “Strongly agree”. Cronbach’s alpha ranged from 0.90 to 0.94.

Furthermore, in this study, empowering leadership is a team-level variable that represents the team members’ collective perception of their team leaders’ empowering style. Thus, the average score for the empowering leadership responses of the team members other than the team leader was used to compute this variable [53]. We performed the following two analyses to validate whether empowering leadership was adequate for aggregation. First, the mean r_wg_ of empowering leadership across the teams (calculated using a uniform null distribution) was 0.83, suggesting a high level of within-team agreement [54]. Second, according to a one-way random-effects analysis of variance, the intraclass correlation coefficient (ICC1) and reliability value of mean group score (ICC2) of empowering leadership were 0.19 and 0.91, respectively. These results suggest that the aggregation of empowering leadership was justified.

Control variables. We controlled for three demographic variables that have been found to influence job crafting [26]. Gender was measured as a dichotomous variable coded as 1 for male and 2 for female. Education was measured using a 5-point scale ranging from “high school level below” to “master’s degree or above”. Tenure was assessed as the number of years worked in the organization and served as a control variable at the individual level (i.e., not at the team-level).

## 4. Results

### 4.1. Preliminary Analyses

Given the multilevel nature of our data, we used hierarchical linear modeling (HLM) analyses from the software HLM 6.06 to test our hypotheses. We first constructed a null model with no predictors and included employees’ job crafting as the dependent variable. The test showed significant results of between-team variances in team members’ job crafting (τ00 = 0.10, χ^2^ = 122.50, df = 63, *p* < 0.05; ICC1 = 0.20, indicating that 20.3% of the variance resided between the teams), justifying HLM as an appropriate analytic technique.

The means, standard deviations, reliabilities, and correlations of the key variables are presented in Table 1.

### 4.2. Hypotheses Testing

The coefficients of the path analysis shown in Table 2 summarize the results of our hypothesis regarding the indirect effect of leaders’ job crafting on team members’ job crafting via leaders’ job resources. Hypothesis 1 predicted that team leaders’ job crafting has a positive effect on team members’ job crafting. The regression analyses of model 1 and model 2 revealed a positive relationship between team leaders’ job crafting and team members’ job crafting after controlling for individual demographic variables at the first level (γ_01_ = 0.39, *p* < 0.001). Thus, Hypothesis 1 was supported. Furthermore, the intergroup variance of team members’ job crafting in model 2 was 0.14 (τ_00_ = 0.05, *p* < 0.05), suggesting that compared with the null model, the proportional reduction in the levels 1 and 2 error variance resulting from the predictors was 33% (PseudoR^2^ = 0.33).

We used the procedure proposed by Preacher and Hayes [55] to examine whether the team leaders’ job resources served as a mediator in the relationship between team leaders’ job crafting and team members’ job crafting (in the teams). According to Preacher and Hayes [55], three criteria should be met to confirm a mediation effect. First, the independent variable should be significantly related to the mediator variable. Second, after controlling for the effect of the independent variable on the dependent variable, the correlation between the mediator variable and dependent variable must be significant. Finally, the indirect effect of the independent variable on the dependent variable must be significant. Before the analyses, all continuous predictors were mean centered [56].

The first step was to regress the mediator (i.e., team leaders’ job resources) on the antecedent (i.e., team leaders’ job crafting). The results revealed a significant relationship between team leaders’ job crafting and team leaders’ job resources (γ_01_ = 0.44, *p* < 0.001). Next, we regressed the dependent variable (i.e., team members’ job crafting) on the mediator (i.e., team leaders’ job resources) while controlling for the antecedent (i.e., leaders’ job crafting). The team leaders’ job resources were significantly related to team members’ job crafting (γ_03_ = 0.35, *p* < 0.001), and the coefficient of the team leaders’ job crafting decreased from 0.39 (*p* < 0.001) to 0.24 (*p* < 0.05). The RMediation program [57] was used to estimate the indirect effects and their 95% confidence intervals. The results showed that the indirect relationship between team leaders’ job crafting and team members’ job crafting was significant (95%, confidence interval CI= 0.05 to 0.26). Therefore, the effect of team leaders’ job crafting on team members’ job crafting was mediated by team leaders’ job resources, and Hypothesis 2 was thus supported. Similarly, we found that the intergroup variance in the team members’ job crafting in model 4 was 0.101 (τ_00_ = 0.04, *p* < 0.05), which implies that compared with model 2, the proportional reduction in the levels 1 and 2 error variance was 26.4% (PseudoR^2^ = 0.26) after adding the mediator to regression model 4.

**Figure 2:**
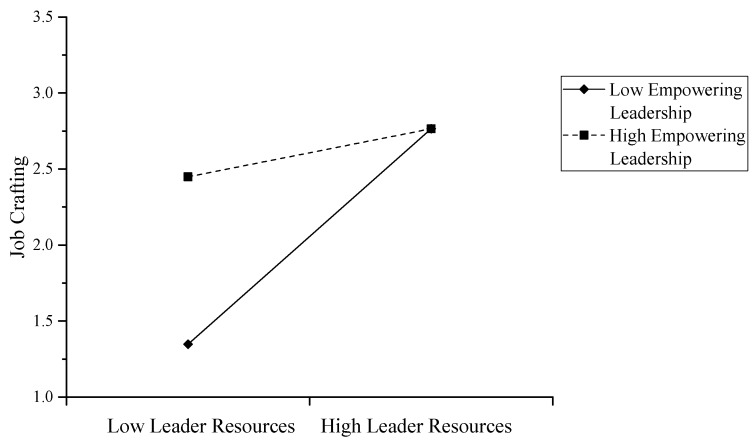
Interaction effect between empowering leadership and leaders’ job resources on team members’ job resources.

Based on model 4, we built model 5 by adding empowering leadership as an interaction term (empowering leadership × team leaders’ job resources) to level 2 of the regression. The results of model 5 are shown in Table 2 and demonstrate that empowering leadership negatively moderates the relationship between team leaders’ job resources and team members’ job crafting (γ_06_ = −0.21, *p* < 0.05). Therefore, Hypothesis 3 was not supported. As shown in Figure 2, the positive relationship between team leaders’ job resources and team members’ job crafting was stronger when empowering leadership was low than when it was high.

**Figure 3:**
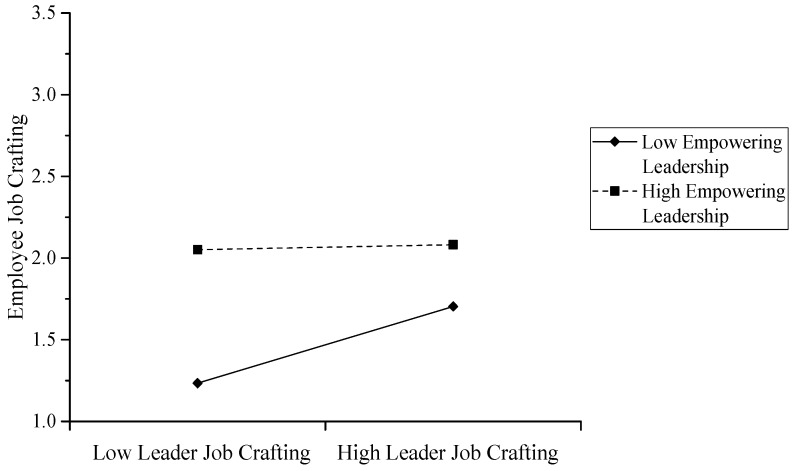
Moderated mediation effect of empowering leadership.

Finally, we examined whether empowering leadership also negatively moderated the indirect effect of team leaders’ job crafting on team members’ job crafting through team leaders’ job resources. We used Mplus7.0 to calculate the direct effects, the indirect effects, and the total effects under the two conditions of one standard deviation below and above the mean of empowering leadership. We then conducted a significance test with model constraint. As shown in Table 3, the indirect effect of team leaders’ job crafting on team members’ job crafting via team leaders’ job resources was significant (γ = 0.21, *p* < 0.01) when empowering leadership was low, but the effect was reduced (γ = 0.10, *p* < 0.05) when empowering leadership was high. Additionally, the difference in the indirect effect of team leaders’ job resources was significant (γ = 0.11, *p* < 0.05). These results indicate that empowering leadership negatively moderates the indirect effect of team leaders’ job crafting on team members’ job crafting via team leaders’ job resources. Furthermore, the product of the coefficient test of the PRODCLIN program [58] confirmed the significance of the indirect effect on team members’ job crafting via team leaders’ job resources with the interaction between team leaders’ job resources and empowering leadership (95% confidence interval CI = (−0.21, −0.05), not containing a zero). Therefore, Hypothesis 4 was not supported. As shown in Figure 3, the indirect effect of team leaders’ job resources was stronger when empowering leadership was low than when empowering leadership was high. 

## 5. Discussion

The purpose of this study was to examine how and when job crafting trickles down from leaders to followers in a team context. Drawing on social learning theory, we proposed that team leaders’ job resources mediate the relationship between team leaders’ job crafting and team members’ job crafting and that empowering leadership further strengthens the relationship between team leaders’ job resources and team members’ job crafting, thereby strengthening the overall mediated relationship (i.e., moderated mediation model). We tested our model with leader-subordinate paired data from 64 work teams in Chinese enterprises over two time periods. Our results show that a team leader’s job crafting is positively related to his/her followers’ job crafting in the team. Team leaders’ job resources were found to mediate the latter relationship. Contrary to our prediction, empowering leadership was found to negatively moderate the relationship between team leaders’ job resources and team members’ job crafting. Furthermore, empowering leadership was also found to negatively moderate the overall mediated relationship. These findings make three important contributions to the job crafting literature. Practical implications are also discussed below.

### 5.1. Theoretical Implications

First, our findings extend the research on job crafting that takes a leadership approach by introducing a trickle-down effect of job crafting from leaders to followers. The extant literature has well documented that several leadership styles, such as transformational and servant leadership [7,11] and several other leadership styles [17,32,59,60], facilitate employee job crafting behavior. However, whether and how job crafting trickles down from leaders to followers in a team context remains unknown. We therefore contribute to this literature by shedding light on the vertical crossover of job crafting from leaders to followers in teams. Moreover, in doing so we demonstrate the importance of role modeling job crafting behavior in teams. Other related studies have already demonstrated that in a team context, leaders influence their followers’ behaviors by role modeling required work behaviors [33,34]. Yet in job crafting research, this has been largely neglected and leadership styles are included as antecedents of employee job crafting behaviors, rather than leader behaviors [6,7,11]. With this research we contribute to a more comprehensive understanding of leadership antecedents in job crafting research. Moreover, the trickle-down effect has been studied in other topics such as abusive leadership [61], work engagement [12], ethical work behavior [13], and deviant work behavior [14], but the present research expends this scope by including job crafting in the trickle-down phenomena.

Second, we introduce social learning theory into job crafting research and further reveal the mechanisms underlying the social learning process in the trickle-down effect of job crafting. Because research demonstrates that job crafting increases job resources [5,9], we examined leaders’ job resources as a mediator explaining why job crafting trickles down from leaders to followers in teams. We therefore also contribute to trickle-down research by introducing another mediator and by responding to the call for more research examining the mechanisms underlying transmission effects [4,11,59]. However, research demonstrates that job crafting also improves performance and increases well-being [9], and such positive outcomes may also be a reason why team members would mimic their leader’s job crafting behavior [15,16]. We therefore encourage future researchers to examine different mediators to further enhance our understanding of the trickle-down effect in job crafting research.

Third, the present research advances our understanding of the boundary conditions of the social learning process that underlies the trickle-down effect of job crafting. Social learning theorists have always shown interest in identifying potential contextual factors that may constrain or facilitate the social learning process [62,63]. By examining empowering leadership as a moderating variable influencing the trickle-down effect, we further clarify the conditions under which job crafting can be transmitted from leaders to followers. Specifically, the results show that empowering leadership weakens the relationship between team leaders’ job resources and team members’ job crafting, and accordingly weakens the overall mediation. This finding contradicts our predictions but may be explained based on prior research suggesting that when employees have access to their leader’s job resources, a leader becomes less necessary [64]. Moreover, when a strong empowering leadership style is adopted, team members may not see a need to perform job crafting behavior because they may already be satisfied with their jobs and work environment. This is consistent with perspectives on leadership and job resources that suggest that empowering leadership may be more effective if required job resources are lacking [65,66]. We highlight there might be a kind of substitution relation between leaders’ job resources and empowering leadership. Nevertheless, when a leader adopts an empowering leadership style, employees are provided with more opportunities to own their jobs [43,44,45], which should further motivate them to create a better job fit by crafting their jobs. We therefore recommend that future studies further unravel this unexpected finding using qualitative research. 

Finally, the present study examined perceptions of empowering leadership at the group level and investigated its impact on individual behavior (i.e., team members’ job crafting). This enabled us to incorporate the team context into the trickle-down effect of job crafting. Although the team context has been incorporated in trickle-down work engagement research [12], we are the first to do so in job crafting research. Moreover, while previous work has extensively studied the role of empowering leadership in enhancing proactive work behaviors [46,65,67,68,69,70], our study is the first to examine its moderating role in a job crafting trickle-down model.

### 5.2. Managerial Implications

Some practical implications can be drawn from the present study. First, team leaders can increase their followers’ job crafting behavior by role modeling the behavior, and team leaders’ job resources play a pivotal role in this process. Therefore, leaders are advised to emphasize to their followers that job crafting produces positive consequences, such as more job resources. Second, a leader can play an active role in further encouraging followers’ job crafting by adopting an appropriate leadership style. When leaders adopt a weaker empowering style, followers will be more encouraged to mimic their leader’s job crafting behavior. Stronger empowering leadership appears to be less effective in this matter. However, because an empowering leadership style is important for employee development [43,44,45], we recommend that leaders find the right balance in adopting such a style without negatively influencing employee development and the job crafting trickle-down effect. Special leader training and development programs can be provided to help leaders achieve this goal.

### 5.3. Limitations and Directions for Future Research

Several limitations of the study should be acknowledged. First, we measured team leaders’ and team members’ job crafting using the same scale. This scale and other job crafting scales do not distinguish between the differences in the job crafting behaviors of leaders versus followers [7,9]. Such a distinction is recommended because leaders have different work responsibilities, and they may therefore craft their jobs differently. This problem raises validity concerns, especially regarding the measurement of team leaders’ job crafting behavior. We recommend that future research develops and validates a job crafting scale to more accurately measure leaders’ job crafting behavior. This would open more doors for empirical research investigating leaders’ job crafting behaviors.

Second, we examined team leaders’ job resources as a mediating variable in our trickle-down model without controlling for other possible mediating situations. According to social learning theory, individuals imitate the behaviors of role models. Although leaders are considered to be role models [3,49], influential peers in a team may also serve as role models. Therefore, an important future direction is to conduct qualitative research to explore the influence of peers in a team environment and to incorporate this as a control variable in future job crafting trickle-down research.

Third, we examined the transmission effect of job crafting between two hierarchical levels (i.e., team leaders and followers). This is in line with prior work examining the trickle-down effect in teams in Chinese enterprises [12]. China has a hierarchical culture characterized by a high-power distance between higher- and lower-ranked employees. Therefore, team leaders have much more authority over followers, and in such a context it is thus appropriate to study a transmission effect between leaders and followers. Nevertheless, the trickle-down effect can occur between three hierarchical levels [13]. Hence, future research is needed to examine our model with a sample including top management, team supervisors, and team members. It would also be worthwhile to replicate our study in other countries to lend further support to our research.

Fourth, we collected data at two time points to test our model. A rigorously longitudinal design with three time points for data collection is needed to infer the causality of the hypothesized relationships. Such a design would allow potential reverse and reciprocal relationships to be included. We recommend that future researchers validate our findings using such a longitudinal design.

Finally, we collected our data only in Chinese science- and technology-oriented organizations, which may limit the generalizability of our research findings. Future studies can replicate our findings using data from other industries. Moreover, the employees were from various sectors, and we failed to control for the differences that may exist in work teams among these sectors. Researchers are highly encouraged to address this shortcoming in future work.

## 6. Conclusions

Although previous studies have shown that leadership is an important antecedent of employee job crafting behavior, no studies have focused on examining how leaders’ job crafting behavior can be transmitted to followers in a team context. The central aim of the present study was to examine when and how job crafting trickles down from team leaders to team members. The results demonstrate that team leaders’ job resources mediate the relationship between team leaders’ job crafting and team members’ job crafting. Empowering leadership was found to further strengthen the mediation relationship more strongly when it was low than when it was high.

## Figures and Tables

**Table 1:** Mean, standard deviations, and correlation matrix.

Variables	M	SD	1	2	3	4	5
**Individual-level**							
1. Age	29.95	7.04					
2. Gender	1.36	0.48	0.18 *				
3. Education	3.70	0.78	−0.03	0.14 *			
4. Tenure	5.84	7.26	0.86 ***	0.14 *	−0.13		
5. Team members’ job crafting	2.34	0.69	−0.05	−0.03	0.09	−0.05	**0.91**
**Team-level**							
1. Leaders’ job crafting	2.50	0.60	**0.92**				
2. Leaders’ job resource	3.80	0.51	0.55 ***	**0.89**			
3. Empowering leadership	4.68	0.49	0.37 ***	0.44 ***	**0.92**		

Note: *n* (individuals) = 250; *n* (teams) = 64. Bold figures on the diagonals are scale reliabilities (Cronbach’s alpha). * *p* < 0.05, *** *p* < 0.001.

**Table 2:** Hierarchical linear modeling (HLM) results: Main and interactive effects.

Variables	Leader’s Job Resource	Team Employees’ Job Crafting			
	Model 3	Model 1	Model 2	Model 4	Model 5
Intercept (γ_00_)	1.99 ***	2.24 ***	1.45 **	0.29	1.76 ***
**Level 1 control variables**					
Gender (γ_10_)		−0.11	−0.10	−0.07	−0.06
Age (γ_20_)		−0.01	−0.01	−0.00	−0.01
Tenure (γ_30_)		0.00	0.01	0.00	0.01
Education (γ_40_)		0.07	0.04	0.07	0.05
**Level 2 independent variables**					
Leaders’ Job Crafting (γ_01_)	0.44 ***		0.39 ***	0.24 *	0.25 *
Empowering Leadership (γ_02_)					0.30 ***
**Level 2 mediated variables**					
Leaders’ Job Resource (γ_03_)				0.35 ***	0.26 *
**Level 2 Cross-level interactions**					
Empowering Leadership × Leaders’ Job Resource (γ_06_)					−0.21 *

Note: *N* (individuals) = 250; *N* (teams) = 64. The coefficients in the table are non-standardized regression coefficients. * *p* < 0.05, ** *p* < 0.01, *** *p* < 0.001.

**Table 3:** Results of moderated mediation effects.

Moderator Variable Empowering Leadership	Stage		Effect		
	First	Second	Direct	Indirect	Total
Low (−1 s.d.)	0.45 ***	0.47 ***	0.23 **	0.21 ***	0.46 ***
High (1 s.d.)	0.45 ***	0.21 **	0.23 **	0.10	0.34 ***
Differences between low and high	0.00	–0.26 ^†^	0.00	–0.11 *	–0.11 *

Note: s.d. = standard deviation. ^†^ 0.05 < *p* < 0.1, * *p* < 0.05, ** *p* < 0.01, *** *p* < 0.001.
